# Enforcement of underage sales laws as a predictor of daily smoking among adolescents – a national study

**DOI:** 10.1186/1471-2458-9-107

**Published:** 2009-04-17

**Authors:** Joseph R DiFranza, Judith A Savageau, Kenneth E Fletcher

**Affiliations:** 1 Department of Family Medicine and Community Health, 55 Lake Avenue, University of Massachusetts Medical School, Worcester, Massachusetts, USA

## Abstract

**Background:**

With a goal to reduce youth smoking rates, the U.S. federal government mandated that states enforce laws prohibiting underage tobacco sales. Our objective was to determine if state compliance with tobacco sales laws is associated with a decreased risk of current daily smoking among adolescents.

**Methods:**

Data on tobacco use were obtained from a nationally representative sample of 16,244 adolescents from the 2003 Monitoring the Future survey. The association between merchant compliance with the law from 1997–2003 and current daily smoking was examined using logistic regression while controlling for cigarette prices, state restaurant smoking policies, anti-tobacco media, and demographic variables.

**Results:**

Higher average state merchant compliance from 1997–2003 predicted lower levels of current daily smoking among adolescents when controlled for all other factors. The odds ratio for daily smoking was reduced by 2% for each 1% increase in merchant compliance. After controlling for price changes, media campaigns and smoking restrictions, a 20.8% reduction in the odds of smoking among 10^th ^graders in 2003 was attributed to the observed improvement in merchant compliance between 1997 and 2003. A 47% reduction in the odds of daily smoking could be attributed to price increases over this period.

**Conclusion:**

Federally mandated enforcement efforts by states to prevent the sale of tobacco to minors appear to have made an important contribution to the observed decline in smoking among youth in the U.S. Given similar results from long-term enforcement efforts in Australia, other countries should be encouraged to adopt the World Health Organization Framework on Tobacco Control strategies to reduce the sale of tobacco to minors.

## Background

Starting in 1987, public health advocates in the U.S. began to pursue a supply-side strategy to reduce smoking among adolescents by making it more difficult for youth to purchase tobacco [1]. Initial efforts to enforce laws that prohibit the sale of tobacco to minors occurred at the community level and consisted of routine inspections of retailers through test purchases conducted by underage decoys [2]. In uncontrolled studies, declines in adolescent smoking of 8%,[3] 26%,[4] 31%,[5] 44%[6] and 50% [2,7] have been reported with interventions to prevent tobacco sales to youth. In controlled studies, communities with strong enforcement have seen relative reductions in youth smoking of 28%, [8,9] 44%, [10] and in three studies, 50% [11-13] and 70% [14]. Seven multivariate studies have shown a favorable impact of youth access laws on adolescent smoking when controlled for price or other tobacco control policies [15-21].

Following reports of substantial reductions in youth smoking achieved by local enforcement [2,5,6], the U.S. federal government enacted the Synar Amendment that requires states to enact and enforce laws prohibiting the sale of tobacco to minors [22]. Although the World Health Organization Framework Convention on Tobacco Control recommends efforts to reduce the supply of tobacco to youth [23], to our knowledge, extensive enforcement efforts have so far been adopted only in the U.S., Canada and Australia, and all three countries have observed substantial and unprecedented reductions in smoking among youth [13,24,25]. Additional strategies to reduce the availability of tobacco to minors include bans on cigarette vending machines and price increases achieved through taxation and litigation. Demand reduction strategies for adolescents include bans on tobacco advertising and promotions, package warning labels, anti-tobacco education and mass-media programs, and efforts to remove smoking imagery from films.

The impact of tobacco sales law enforcement has never before been evaluated in a national study, although successful results from a long-term regional study in Australia are in press [13]. Prior to the Synar Amendment going into effect in 1996 [26], compliance with tobacco sales laws was uniformly poor across the U.S. [27]. Subsequent to the Synar mandate, states instituted enforcement involving test purchases (compliance tests) and fines [28]. Mandatory annual state progress reports document marked improvement in compliance in 49 states between 1997 and 2003 [29]. Over these years, the prevalence of daily smoking among 10^th ^graders fell 51% nationwide from 18.0% to 8.9% [30]. At the same time, states began to adopt clean indoor air policies [31], the tobacco industry settled a large suit and raised prices [32,33], and a national anti-tobacco media campaign was launched in 2000 [34]. The purpose of this study was to determine if average state-level compliance from 1997 to 2003 predicts current daily smoking among 10^th ^graders in 2003, while considering the effects of price, clean indoor air policies, and anti-tobacco media.

## Methods

We studied the natural experiment resulting from geographic variations in youth's exposure to interventions. Prospectively collected compliance data from 1997–2003 was used with data on price, smoking policies, and a media campaign to determine the impact of each on the odds that a youth was a daily smoker while controlling for age, gender, race, Hispanic ethnicity and parental education.

### Population

We used the most current data (spring of 2003) from the Monitoring the Future study (MTF), an annual survey conducted in compliance with human subjects protections [30]. Thirty-six states were included in a multi-stage proportional sampling strategy with weighting to render a nationally representative sample [30]. It should be noted that the MTF sample is not representative of states and state is not a cluster factor in the sample design. The survey was completed by 16,300 10^th ^graders (age range 15–17 years) reflecting a participation rate of 88%. Sociodemographic information was available for state of residence, and student-reported measures of age, gender, race, ethnicity, and parental education. Race was dichotomized as white/nonwhite and ethnicity as Hispanic/non-Hispanic. The MTF database includes a parental education variable that is based on the average of maternal and paternal education coded into 11 levels. As the MTF data do not measure smoking prevalence by state, a state-level analysis was not possible. For each state, data concerning merchant compliance, cigarette prices, restaurant smoking policies, and anti-tobacco media for each year from 1997 to 2003 were obtained.

### Outcome Variables

Although the MTF survey also includes 8^th ^and 12^th ^graders, we chose to focus on 10^th ^graders based on the consideration that very few 8^th ^graders purchase tobacco, and by the 12^th ^grade most youth can legally purchase it [8]. We considered ever having tried smoking and current daily smoking (smoking in the previous 30 days) as outcome variables. Only 1% of youth purchase their first cigarette, so enforcement would impact ever having tried smoking only indirectly and we did not expect positive results [35]. Among current smokers, those who smoked daily would be the most directly affected by enforcement programs since youth do not typically begin to purchase their own tobacco until they are daily smokers (smoking at least one cigarette per day) [8].

### State Compliance Rates

The Synar Amendment requires states to conduct annual surveys to measure merchant compliance [26]. The surveys are conducted by sending underage decoys into stores to attempt to purchase tobacco. The surveys include a representative statewide sample of tobacco retailers and produce a compliance rate which indicates the percentage of merchants in compliance (with a 95% confidence interval of no more than +/- 3%). State protocols vary in aspects that influence the measured rate of compliance such as the age of the youths and whether they offer proof of age [36-38]. The first year in which all states collected compliance data was 1997 [39]. Compliance data were obtained from the Center for Substance Abuse Prevention [39]. Eight compliance variables were created: 7 for the federal fiscal years 1997–2003, and 1 for the 7-year average.

### Price

The Tax Burden on Tobacco provides quarterly prices (including taxes), by state, adjusted for inflation to 1983 dollars [32]. Since youths rarely purchase generic brands,[35] the price with generics excluded was used. The average price was computed for the 12 months preceding the MTF survey in the 2^nd ^quarter of each year. Eight variables were created, one for each year from 1997–2003, and one for the 7-year average.

### Restaurant Smoking Policies

State restaurant smoking policy data were obtained from the Centers for Disease Control and Prevention [31]. Too few states had banned restaurant smoking by 2003, so a policy variable was created for each year, such that any statewide restriction on restaurant smoking was assigned a value of 1 and no restrictions, a zero. An eighth variable represented the 7-year average. We did not include local restrictions on restaurant smoking as only 1% of the national population were covered by such ordinances during the study period (compared to over 50% for state level regulations), and the completeness of the available databases could not be verified [40].

### Anti-Tobacco Media Exposure

The American Legacy Foundation graciously shared data concerning expenditures in each of 200 designated market areas in the U.S. for their national anti-tobacco media campaign for each year from 2000 to 2003. Market buys were used as an indirect measure of exposure to the campaign. Four variables were computed representing spending in each of the four years, plus a fifth variable, the four-year average.

### Statistical Analyses

For all analyses the outcome was smoking by individual subjects (ever used tobacco, or daily smoking). Subjects were assigned values for compliance, price, policy and media variables based on the location of their school. Age, gender, race, ethnicity and parental education (demographic variables) were forced into each logistic regression analysis as independent variables because these have each been identified as smoking risk factors in prior studies, because parental education is a good proxy for parental smoking status, and because states vary on several of these factors [41]. Regressions were initially run for each annual compliance, price, policy, and media variable individually. The 7 annual variables for compliance, or price, or policy, and the 4 annual variables for media could not be included together because values from one year to the next were highly correlated and this created issues with multi-colinearity. For example, almost all states had the same restaurant smoking policy for each of the 7 years. We next conducted a separate regression for each multi-year average variable. As none of the media or policy variables were significant at p < 0.20 in the preceding models, they were not included in the final model. Thus, the final model included 'average compliance', 'average price' and the sociodemographic variables. All results were weighted to provide a nationally representative sample. Analyses, using Proc SurveyLogistic in SAS,[42] included clustering by school (as state was not a cluster variable in the MTF sampling strategy).

### Ethics

Our analysis was exempt from ethical review as it was a secondary analysis of de-identified data. The MTF study is conducted in compliance with ethical approval from the University of Michigan.

## Results

There were 16,244 subjects of whom 99.4% were between 15 and 17 years of age (range = 10–18; mean = 15.6); 51.4% were female; 62.8% White, 16.7% Black, 11.6% Hispanic and 8.9% other races. Smoking in the past 30 days was reported by 16.7% and current daily smoking by 8.9%.

The proportion of subjects in states with restaurant policies was 52.9% in 1997 and 53.8% in 2003. Between 1997 and 2003, the population-weighted mean compliance rate for the 36 MTF states increased from 77.2% to 87.6%, the standard deviation (SD) decreased from 8.76 to 4.61, and the range decreased from 53.3 to 19.9, reflecting decreased variability between states. Over the same years, the population-weighted mean inflation-adjusted price increased from $1.27 to $2.21 in 1983 dollars, the SD increased from $0.17 to $0.32, and the range grew from $0.74 to $1.38, reflecting increasing variability.

When average compliance, average price and average restaurant policy were included in a single model, the impact of compliance on having ever used tobacco was not significant (p = 0.07) even prior to adjusting for clustering, so subsequent analyses were limited to current daily smoking. With all demographic variables included in one model, the odds of current daily smoking was related to increased age (OR = 1.40, 95% CI: 1.28–1.56, p < 0.0001), decreased parental education (OR = 0.96, 95% CI: 0.95–0.96, p < 0.0001), and white race (OR = 2.29, 95% CI: 1.95–2.70, p < 0.0001), but not to gender or Hispanic ethnicity. The 7-year average compliance from 1997–2003 predicted daily smoking in 2003 (OR = 0.98 for each 1% increase in compliance, 95% CI: 0.96–0.99, p = 0.04). The 7-year average price was also a significant predictor (OR = 0.49 for each dollar increase in price, 95% CI: 0.29–0.83, p = 0.007). Average policy and media were not significant predictors of daily smoking among 10^th ^graders. When both compliance and price were included together in the final model (table 1; adjusting for age, gender, race, ethnicity and parental education), the results did not differ from the models that considered these variables separately.

**Table 1:** The effect on current daily smoking of average compliance and price from 1997–2003 in a multiple logistic regression adjusting for age, gender, race, ethnicity, and parental education, in a U.S. national survey, 2003.

	OR*	95% CI	p
Average Compliance 1997–2003	0.98	.96–.99	0.04
Average Price 1997–2003	0.55	.37–.90	0.02
Age	1.38	1.2–1.6	<0.0001
White race	2.3	1.8–2.8	<0.0001
Parental education	0.96	.95–.97	<0.0001

* Odds Ratios for each 1% increase in merchant compliance, for each $1.00 increase in inflation-adjusted price, and for each year of age. Average policy was not a significant predictor. Intercept -3.48, p = 0.025.

### Population impact

We performed a calculation to determine how daily smoking rates would have been impacted by changes in price and compliance that occurred from 1997 to 2003. The odds of current daily smoking declined by 2% for each 1% increase in average compliance, and declined by 1% for each two cent increase in average price. Using state adolescent census data, we computed that the population-weighted average compliance increased by 10.4 percentage points from 1997 to 2003. Given a 2% decrease in the risk of smoking for each 1% increase in compliance, we calculate that the odds of daily smoking were reduced by an estimated 20.8% due to improved compliance. At the same time, the population-weighted price increased by 94 cents, reducing the odds of daily smoking by 47%. So it can be estimated that under the compliance and price conditions pertaining in the U.S. from 1997–2003, price increases had twice the impact as changes in compliance, but both factors likely contributed to the lower smoking rates observed in 2003.

## Discussion

This analysis of national data covering 7 years indicates that improvements in merchant compliance that were measured in the U.S. from 1997–2003 as states complied with the Synar Amendment predicted a 20.8% reduction in daily smoking among 10^th ^graders in 2003. Prior to the Synar Amendment, compliance with tobacco access laws was quite low [27]. Although a few states acted of their own initiative, there is no doubt that the Synar Amendment with its threat of financial penalties for states was the prime motivator for most states to enforce their laws [26,29]. This is the first study to document that the Synar Amendment achieved its intended effect of reducing adolescent smoking.

The current study adds to a substantial body of evidence that demonstrates that improvements in merchant compliance are associated with reduced daily smoking among adolescents [43]. This is the first study to demonstrate an effect of enforcement of tobacco sales laws on a national level. This is important because of concerns that only isolated rural communities could be successful in this regard. This U.S. national study demonstrates that this approach can be effective across an area encompassing wide geographical and demographic variety.

Several early attempts to reduce youth's access to tobacco failed. Three studies of local ordinances found no impact on youths' smoking [44-46], but these concerned very short-lived, local interventions that failed to convince merchants to obey the law. Other interventions discouraged smoking only among the youngest youth who have the most difficulty purchasing tobacco [45,47,48]. Because the positive and negative studies implemented different types of intervention, the positive studies are not canceled out by the negative studies. With so many positive studies as cited in the background section, there is no question that youth access interventions are capable of reducing smoking among youth. The important research questions concern what are the crucial components of an effective intervention? To date, all successful enforcement programs have relied upon the use of underage decoys to attempt to purchase tobacco [28]. No other method of enforcement has demonstrated effectiveness.

Inaccuracy in the measurement of compliance results in misclassification of exposure status, increasing the risk of failing to detect a real effect. Thus, the fact that compliance checks conducted by decoys are a poor measure of the ability of real underage smokers to purchase tobacco,[49] that states used different aged youth and different protocols to measure compliance, that states have used 14 different protocol procedures that might bias their surveys to produce artificially high compliance rates,[50] and that statewide compliance rates mask substantial variability in the compliance rates experienced by youths living in different communities within a state, all worked to obscure the association between true compliance and youth smoking.

We did not see an impact of compliance on ever having tried tobacco which declined by 28% between 1997 and 2003, about half the decline of 51% observed for daily smoking [24]. Daily smokers are more likely to purchase their own tobacco and would therefore be the primary target for restrictions on tobacco sales [8]. The lower rate of decline in ever trying tobacco is consistent with the lack of an observed impact from enforcement. Novice smokers obtain their cigarettes by begging from friends and do not typically spend their own money purchasing cigarettes until they feel a need to smoke every day. Daily smokers make most tobacco purchases and win friends by supplying peers with cigarettes. Thwarting the sale of tobacco to youth affects daily smokers directly and nondaily smokers mostly indirectly as daily smokers become less willing to share when it becomes difficult for them to purchase [8,51].

Several study limitations must be noted. The absence of evidence for an impact of the media campaign may result from design limitations as others have seen an impact from this campaign using a well-controlled prospective design [34]. We were unable to control for state expenditures on anti-tobacco programs other than Synar compliance, because no state dichotomizes tobacco control funding as Synar/nonSynar. Controlling for total state tobacco control funding without excluding funding for Synar compliance would produce an obvious confound with state compliance rates. The survey relied on self-reported tobacco use. Our prior studies have shown excellent reliability in self-reported cigarette smoking among U.S. adolescents [52]. Further limitations were our inability to control for local programs or tobacco policies. The measure of media exposure did not capture local or state programs. Since this was a national study, it is unlikely that isolated local programs could have impacted the results.

An unidentified factor associated with both merchant compliance rates and youth smoking rates could produce confounding. If anti-tobacco states were faster, or tobacco-growing states were slower to implement Synar, merchant compliance might be a proxy for attitudes toward smoking. A state-by-state review of factors associated with compliance with the Synar Amendment from 1995–2004 did not identify any sources of confounding [28]. Price is a barometer of state tobacco sentiment and the impact of compliance was absolutely unchanged when controlled for price.

Important strengths of this study include the large nationally representative sample, and the long period over which the impact of compliance was assessed. This is important as several years of impaired access may be necessary before effects are evident among older adolescents [12]. The adjustment for price, restaurant smoking policies, anti-tobacco media campaigns, age, gender, race, ethnicity and parental education are additional strengths.

Enforcement carries a resource cost, so it is important to consider whether the benefits outweigh the costs. The cost per year of life saved for youth access enforcement in the U.S. has been estimated at $660 per year of life saved (discounted at 3% annually) for a program that costs $150/retailer per year and results in a 10% reduction in the prevalence of smoking [53]. For comparison purposes, Woodridge, Illinois reported an annual cost of enforcement of $16/retailer per year [53]. The actual annual cost to states of implementing the Synar Amendment is unknown even to them, [53] but if we allow for a generous education and enforcement budget of $150/tobacco retailer for a state-run program, our calculated 20.8% reduction in the odds of current smoking equates to a cost of $330 per year of life saved. This compares favorably to the cost of colorectal cancer screening at $10,000–$25,000,[54] or annual mammography for women ages 40 to 80 at $40,000 per year of life saved, all discounted at 3% annually [55]. For developing countries with limited resources, enforcement of tobacco sales laws presents an attractive, low-cost approach to saving lives. Almost the entire cost of an enforcement program is labor, so in countries with lower labor costs than the U.S., the expenses will be much lower than those used for these calculations.

## Conclusion

Our data indicate that improving merchant compliance with the prohibition on sales of tobacco to minors and increasing the price of cigarettes discourage youth smoking. Our data suggest that an absolute increase in compliance of 25 percentage points has about the same deterrent effect as increasing the price by $2.00 in 2006 dollars ($1.00 in 1983 dollars). But there is no reason why policy makers should choose between these approaches, as all effective measures to reduce smoking among youth should be employed. The revenue generated by a two-cent per pack tax on cigarettes would be sufficient to fully fund a comprehensive enforcement program [53]. The World Health Organization Framework Convention on Tobacco Control recommends that states raise prices and enforce restrictions on the sale of tobacco to minors [23]. Ours is the first study to demonstrate that such a program can be effective on a national level to reduce smoking.

## Competing interests

The authors declare that they have no competing interests.

## Authors' contributions

JRD conceived of the study, obtained funding and wrote the manuscript. JAS and KEF supervised the data analysis and contributed to editing the manuscript.

## Pre-publication history

The pre-publication history for this paper can be accessed here:


